# Enhanced Autophagy Contributes to Reduced Viral Infection in Black Flying Fox Cells

**DOI:** 10.3390/v11030260

**Published:** 2019-03-14

**Authors:** Eric D. Laing, Spencer L. Sterling, Dawn L. Weir, Chelsi R. Beauregard, Ina L. Smith, Sasha E. Larsen, Lin-Fa Wang, Andrew L. Snow, Brian C. Schaefer, Christopher C. Broder

**Affiliations:** 1 Department of Microbiology & Immunology, Uniformed Services University of the Health Sciences, Bethesda, MD 20814, USA; spencer.sterling.ctr@usuhs.edu (S.L.S.); dawn.l.weir.mil@mail.mil (D.L.W.); chelsi.beauregard.ctr@usuhs.edu (C.R.B.); brian.schaefer@usuhs.edu (B.C.S.); christopher.broder@usuhs.edu (C.C.B.); 2 Infectious Diseases Directorate, Naval Medical Research Center, Silver Spring, MD 20910, USA; 3 Risk Evaluation and Preparedness Program, Health and Biosecurity, CSIRO, Black Mountain 2601, Australia; Ina.smith@csiro.au; 4 Department of Pharmacology & Molecular Therapeutics, Uniformed Services University of the Health Sciences, Bethesda, MD 20814, USA; Sasha.Larsen@idri.org (S.E.L.); andrew.snow@usuhs.edu (A.L.S.); 5 Programme in Emerging Infectious Diseases, Duke-National University Singapore Medical School, Singapore 169857, Singapore; linfa.wang@duke-nus.edu.sg

**Keywords:** autophagy, viruses, bats

## Abstract

Bats are increasingly implicated as hosts of highly pathogenic viruses. The underlying virus–host interactions and cellular mechanisms that promote co-existence remain ill-defined, but physiological traits such as flight and longevity are proposed to drive these adaptations. Autophagy is a cellular homeostatic process that regulates ageing, metabolism, and intrinsic immune defense. We quantified basal and stimulated autophagic responses in black flying fox cells, and demonstrated that although black flying fox cells are susceptible to Australian bat lyssavirus (ABLV) infection, viral replication is dampened in these bat cells. Black flying fox cells tolerated prolonged ABLV infection with less cell death relative to comparable human cells, suggesting post-entry mechanisms interference with virus replication. An elevated basal autophagic level was observed and autophagy was induced in response to high virus doses. Pharmacological stimulation of the autophagy pathway reduced virus replication, indicating autophagy acts as an anti-viral mechanism. Enhancement of basal and virus-induced autophagy in bat cells connects related reports that long-lived species possess homeostatic processes that dampen oxidative stress and macromolecule damage. Exemplifying the potential that evolved cellular homeostatic adaptations like autophagy may secondarily act as anti-viral mechanisms, enabling bats to serve as natural hosts to an assortment of pathogenic viruses. Furthermore, our data suggest autophagy-inducing drugs may provide a novel therapeutic strategy for combating lyssavirus infection.

## 1. Introduction

Bats, order Chiroptera, host a significantly greater richness of virus species compared to other mammalian taxa [1]. Viruses hosted by bats include several that are highly pathogenic in human hosts such as henipaviruses (e.g., Nipah virus (NiV) and Hendra virus (HeV)) [2], filoviruses (e.g., Marburg virus (MARV)) [3,4], coronaviruses (e.g., SARS-like coronavirus) [5], and lyssaviruses (e.g., Rabies virus (RABV)) [6]. Bats experimentally infected with NiV and HeV [2,7,8], Ebola virus (EBOV) [9], and MARV [3,4,10], seroconverted, but did not develop pathology and only a minority of infected bats shed low level of virus in urine (NiV) [8] or high levels of virus orally (MARV) [4]. This combination of host–viral richness and apparent lack of clinical disease presentation after NiV, HeV, EBOV, or MARV infection raises the question how novel intrinsic traits or host adaptations might promote tolerance of virus infection in bats [11,12].

As bats are the only volant mammals, flight has been proposed to contribute to the ability of bats to function as natural reservoirs [11,12,13]. The aerobic demands of flight experienced by bats is higher than the aerobic demands of other terrestrial mammals experienced during aerobic exertion [14]. Positive selection of genes involved in DNA-damage repair pathways [11] and reduced free-radical production [15] in bats suggest an evolved response to the deleterious effects of an increased generation of reactive oxygen species (ROS) from the high aerobic demands of flight. Cellular ageing occurs through a progressive accumulation of damaged organelles, misfolded and aggregated proteins, and dysregulation of cellular homeostatic processes [16,17]. ROS can cause oxidative damage to macromolecules and DNA [18], and ultimately contribute to cellular ageing. However, bats have a greater longevity [19] than what is typically projected in relation to their body size [20,21], and resistance to oxidative stress has been observed in long-lived bat species [22,23]. Furthermore, the loss of DNA sensors in the PYHIN family [24] and decreased activity of stimulator of interferon genes (STING) [12], may contribute to dampened inflammatory responses to the release of cytosolic DNA due to metabolic-related DNA damage or microbial infection [25].

In addition, enhanced protein homeostatic mechanisms, such as macroautophagy (hereafter referred to as autophagy), were observed in bat cells [26]. At the crossroads of DNA-damage, oxidative stress, and microbial infection [27,28], autophagy, is a proteostatic process involved in cellular homeostasis, longevity, and survival [29,30]. Canonically regarded as a response to starvation, the autophagy pathway recycles long-lived proteins, misfolded proteins, and damaged organelles during times of nutrient depletion. However, there are also multiple examples of selective autophagic pathways that utilize specific protein receptors to traffic ubiquitinated target organelles and proteins into cytoplasmic double-membraned vesicles, termed autophagosomes [31]. In addition to cellular stressors such as DNA damage and ROS, pathogen-recognition receptors can activate distinct autophagic processes as part of an intrinsic immune response [32,33,34]. For example, “xenophagy” shuttles intracellular microbes to autolysosomes for lysis, whereas “aggrephagy” removes toxic protein aggregates [35,36].

Aerobic demands, ROS, nutritional starvation, or virus infections are all physical stressors that challenge cellular homeostasis [37]. Virus exploitation of host cell transcriptional and translational machinery ensures successful replication, but can trigger the unfolded protein response [38], and dysregulation of cellular homeostatic mechanisms [39]. Therefore, if bats have evolved mechanisms to dampen cellular damage caused by the by-products of heightened aerobic respiration through improved proteostatic mechanisms, then these same proteostatic mechanisms might also function as anti-viral defenses.

We undertook a comparative study to examine host–pathogen interactions in bat versus human cells, focusing on the potential anti-viral or pro-survival mechanism of autophagy. For this study we chose to utilize Australian bat lyssavirus (ABLV), a neurotropic rabies-related virus that naturally infects black flying foxes (*Pteropus alecto*) [40,41], but is classified as a biosafety level 2 (BSL-2) pathogen. *Pteropus* species are also the natural reservoirs of several zoonotic viruses including HeV, NiV [2], and Menangle virus [42,43]. Cell lines have been established from the black flying fox [44], which with the publication of its reference genome [11], has been promoted as a model bat species. Black flying fox cell lines have been used to investigate the interferon response [45,46,47,48,49,50], as well as transcriptomic and proteomic responses after HeV infection [51]. We rescued a modified recombinant ABLV expressing a green fluorescent protein (rABLV-GFP) and used both rABLV-GFP and a wild-type ABLV (wt-ABLV) to examine the role of autophagy after virus infection. In black flying fox cells, the basal level of autophagy was significantly higher than the levels of autophagy quantified in the human cell line used for comparative purposes. We observed that ABLV infection activated the autophagy pathway in a dose-dependent manner, in both black flying fox- and human-derived cell lines, which we confirmed in primary black flying fox brain cells. Activation of autophagy through pharmacological methods decreased ABLV replication in both black flying fox and human cells, which suggested (1) that autophagy functions as an anti-viral defense during ABLV infection, and (2) that activation of autophagy might be an effective treatment against neurotropic viruses such as ABLV or related lyssaviruses. Lastly, we observed that in contrast to human cells, black flying fox brain-derived cells withstood a high dose of ABLV over a long incubation period and experienced significantly less cell death. Our findings provide an initial in vitro exploration for future studies that may illuminate the importance of autophagy as an enhanced post-transcriptional anti-viral pathway in bats.

## 2. Materials and Methods

### 2.1. Cells and Viruses

Black flying fox brain (PaBrH) and kidney (PaKiT) tissue-derived cell lines and primary brain (PaBr) cells have been previously described [44]. PaBrH and PaKiT cells were maintained in DMEM (Dulbecco’s Modified Eagle Medium (Gibco Laboratories; Gaithersburg, MD, USA), 10% HyClone^™^ Cosmic Calf^™^ Serum (CCS) (Thermo Fisher Scientific; Waltham, MA, USA), and 1% l-glutamine (Thermo Fisher Scientific)) complete cell culture media (DMEM-10). Primary PaBr cells were maintained in DMEM/Nutrient F-12 Ham media (Sigma-Aldrich; St. Louis, MO, USA) with 10% fetal bovine serum (Gibco) and 1% Antibiotic-Antimycotic (Gibco). A human neuroblastoma cell line (NBF-L) was obtained from Dr. Aviva Symes (Uniformed Services University, Bethesda, MD, USA) and maintained in DMEM supplemented with 10% Cosmic Calf^™^ Serum (Thermo Fisher Scientific), 5% fetal bovine serum (Gibco), and 1% GlutaMAX^™^ (Thermo Fisher Scientific). Human embryonic kidney (HEK) 293T (ATCC^®^ CRL-3216^™^) and mouse Neuro-2a (ATCC^®^ CCL-131^™^) cells were obtained from the American Type Culture Collection (ATCC; Manassas, VA, USA) and maintained in DMEM-10 complete media. A recombinant Australian bat lyssavirus (rABLV), human isolate [52], anti-genome plasmid was used to generate a reporter virus through reverse genetics and a wild-type ABLV (wt-ABLV), *Pteropus* isolate [40], was also used for infection studies (NCBI GenBank accession number: AF418014).

### 2.2. Rescue of Recombinant ABLV-GFP Reporter Virus

The open reading frame of Turbo green fluorescent protein (GFP; Evrogen; Moscow, Russian Federation) was cloned into the rABLV anti-genome plasmid between the ABLV *glycoprotein* (*G*) and *large RNA polymerase* (*L*) genes. To rescue rABLV-GFP, HEK293T cells were seeded 2 × 10^5^ cells/well in 6-well plates and transfected with 2 µg full-length rABLV-GFP anti-genome plasmid and 1 µg nucleoprotein (N), 0.5 µg phosphoprotein (P), and 0.5 µg L protein helper plasmids. The total recombinant plasmid DNA (4 µg) was mixed with 10 µL Lipofectamine LTX^®^ (Thermo Fischer Scientific) in OPTI-MEM (Gibco), following standard transfection protocol. HEK293T cells were maintained in DMEM-10 complete media. Cell culture supernatants were replaced 24 hours post-transfection with DMEM, 6% CCS and 1% l-glutamine. Three days post-transfection, fluorescent microscopy was used to examine GFP expression. Wells that contained GFP-positive cells were passaged and co-cultured with mouse Neuro-2a cells. Cells were continuously monitored for GFP expression and cell culture supernatant was passaged to fresh HEK293T cells. To purify rABLV-GFP, cell culture supernatant containing rABLV-GFP was collected and ultracentrifuged (27,500 rpm, 2 h) with a 20% sucrose cushion.

### 2.3. ABLV Infections and Immunoblots

PaBrH, PaKiT, HEK293T, NBF-L, or primary PaBr cells (2.5 × 10^5^ cells/well, 6-well cell culture plates) were infected with rABLV-GFP or wt-ABLV at the indicated multiplicities of infection (MOI) (e.g., 0, 1.0, 10). To compare replication kinetics between PaBrH, PaKiT, HEK293T, and NBF-L, cell lines were incubated with 1 mL DMEM-10 containing rABLV-GFP (MOI 1) for 2 h. After 2 h, virus/DMEM-10 was removed and replaced with fresh DMEM-10. Cell culture supernatants were collected and centrifuged to remove cell debris (2500 rpm, 10 min) then serially diluted and incubated with HEK293T cells to determine ABLV titers. GFP expression from rABLV-GFP was used to count the number of fluorescent virus plaques, whereas FITC-conjugated anti-Rabies monoclonal globulin (Fujirebio Diagnostics, Inc.; Malvern, PA, USA) was used to quantify wt-ABLV titers. To quantify GFP expression by flow cytometry (LSR II; BD Biosciences; San Jose, CA, USA), cell culture supernatants were collected and centrifuged to collect floating cells, then mixed with the collected adherent cells, and fixed with 4% paraformaldehyde. GFP median fluorescence intensity (MFI) and the percentage of GFP positive cells were then analyzed.

Bafilomycin A1 (BAFA1; 400 nM; InvivoGen; San Diego, CA, USA) was used to inhibit fusion of autophagosomes and autolysosomes, i.e., autophagic flux. To block autophagy, cells were incubated for two hours at the end of the infection time course with BAFA1 prior to whole cell lysate processing. Whole cell protein lysates were collected at indicated hours post-infection (hpi) and processed following protocol guidelines for autophagic quantification by LC3B western blots [53]. Cell culture protein lysates were normalized to 15 µg, and loaded into 4–12% Bis-Tris protein gel (Invitrogen; Carlsbad, CA, USA); protein gels were transferred to PVDF membranes (Bio-Rad; Hercules, CA, USA; 350 mA, 2 h) for standard western blotting techniques. Antibodies used for immunoblots included: anti-LC3B polyclonal antibody (Sigma-Aldrich), anti-p62 polyclonal antibody (Sigma-Aldrich), anti-NDP52 polyclonal antibody (Aviva Systems Biology; San Diego, CA, USA), anti-β-actin polyclonal antibody (Abcam; Cambridge, UK), anti-GAPDH polyclonal antibody (Abcam), and anti-Turbo GFP polyclonal antibody (Evrogen). An anti-Rabies virus N protein polyclonal rabbit serum was provided by Dr. Ina Smith and anti-Rabies virus P protein polyclonal antibody was purchased (MyBioSource; San Diego, CA, USA). Protein levels were quantified using Image J software (NIH; Bethesda, MD, USA).

### 2.4. LIVE/DEAD Violet Cell Staining

PaBrH and NBF-L cell lines at a density of 2.5 × 10^5^ cells/well (6-well plate) were infected with rABLV-GFP. Cell culture supernatant was not removed after the addition of rABLV-GFP until the conclusion of the post-infection time course. Cell culture supernatant and cells were collected, centrifuged for 5 min, 2500× *g*, resuspended in 1 mL PBS, and dyed with LIVE/DEAD™ Fixable Violet Dead Cell Stain Kit, for 405 nm excitation (Thermo Fisher Scientific) for 30 min following the manufacturer’s guidelines. Cells were fixed with 4% paraformaldehyde and incubated at 37 °C for 20 min. Cells were then pelted and washed with PBS, this process was repeated twice. Resuspended stained cells were analyzed with an LSR II Flow Cytometer (BD Biosciences) for ABLV GFP expression and LIVE/DEAD Violet signal.

### 2.5. Pharmacological Activation of Autophagy

PaBrH, PaKiT, and NBF-L cells at a density of 2.5 × 10^5^ cells/well (6-well plate) were infected with rABLV-GFP (MOI 1.0) for 48 h. At 24 hpi, rapamycin (RAPA; Sigma-Aldrich), small molecule enhancer of autophagy-28 (SMER; TOCRIS; Bristol, UK), or NVP BEZ235 (BEZ; Selleck Chemicals; Houston, TX, USA) were added to the wells and incubated with the ABLV-infected cell culture for 24 h. At 48 hpi, cell culture supernatants and whole cell lysates were collected. Whole cell lysates were processed as described above. Supernatants were centrifuged for 10 min at 2500 rpm to remove cell debris, then serially diluted and incubated with HEK293T cells at 5 × 10^4^ cells/well (96-well plate) to determine rABLV-GFP titers; FITC anti-Rabies monoclonal globulin (Fujirebio Diagnostics) was used to quantify wt-ABLV titers after BEZ treatments. We attempted to monitor autophagy levels by flow cytometry after RAPA and SMER induction using a CYTO-ID^®^ Autophagy detection kit (Enzo Life Sciences, Inc.; Famingdale, NY, USA), which stains autophagosomes with a green fluorescent dye; a positive stain was confirmed using the PaKiT cell line.

## 3. Results

### 3.1. ABLV Replication in Black Flying Fox Cell Lines

**Figure 1:**
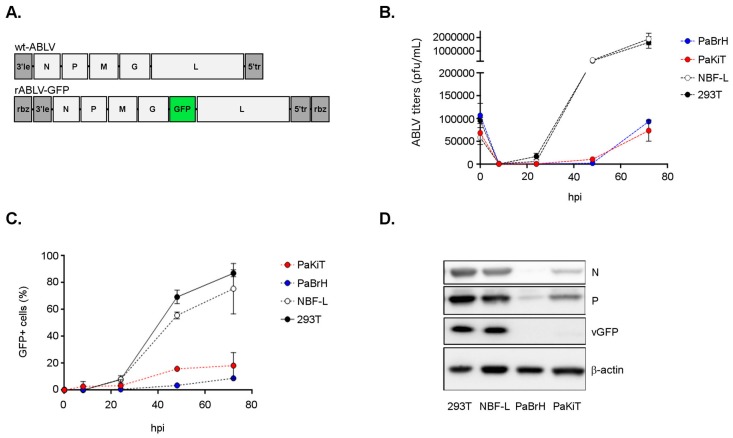
ABLV replication is reduced in black flying fox cells. (**A**) wt-ABLV and rABLV-GFP genome schematics. (**B**) rABLV-GFP titers. Cells were incubated with rABLV-GFP (MOI 1) then after two hours the virus inoculated cell culture supernatant was replaced with fresh cell culture media. Cell culture supernatant and cells were collected after at indicated hpi and serial dilutions were incubated with fresh HEK293T cells to determine titers. (**C**) Flow cytometry analysis of GFP positive cells over a time course of rABLV-GFP infection. (**D**) Western blot image of ABLV N, P, and vGFP expression in all cell lines at 72 hpi. Data are a representation of two independent experiments, mean ± SEM.

ABLV, like all lyssaviruses, has a 3’-5’(-)ssRNA genome that encodes five proteins: nucleoprotein (N), phosphoprotein (P), matrix protein (M), envelope glycoprotein (G), and large RNA polymerase (L). We used reverse genetic techniques to generate a recombinant ABLV reporter virus expressing a green fluorescent protein (rABLV-GFP) (Figure 1A). The ABLV anti-genome plasmid was modified with the insertion of a GFP open reading frame between the *G* and *L* genes. First, we compared ABLV replication in black flying fox and human cell lines. To conduct these experiments, we utilized brain (PaBrH) and kidney (PaKiT) tissue-derived black flying fox cell lines [44]. The PaBrH cell line is morphologically fibroblast-like in appearance so we chose to compare ABLV replication with a human neuroblastoma cell line (NBF-L) that also had fibroblast-like morphology [54]. A human embryonic kidney cell line, that was similarly immortalized with a SV40 T antigen (HEK293T), was included for comparative purposes with the PaKiT cell line.

Lower rABLV-GFP titers were quantified from both infected black flying cell lines compared to human cell lines at equivalent hpi (Figure 1B). Nearly 10-fold higher titers were achieved in both human compared to black flying fox cell lines at 48 and 72 hpi. Quantification of GFP positive cells after rABLV-GFP infection revealed that nearly double the number of human cells were infected compared to black flying fox cell lines at 48 and 72 hpi (Figure 1C). Investigation of ABLV protein levels—including nucleoprotein (N), phosphoprotein (P), and the virus-expressed GFP (vGFP) (Figure 1D)—revealed lower amounts of structural and non-structural ABLV proteins in black flying fox cells. GFP expression in black flying fox cells and human cells indicated that all four cell lines were susceptible to rABLV-GFP infection, although multiple mechanisms of cellular entry, replication, or post-entry might contribute to the reduced level of replication in black flying fox cells relative to human cells. To avoid issues with inefficient ABLV entry into black flying fox cells, in the following experiment the conditions of rABLV-GFP infection were modified to promote infection of close to 100% of the cells.

### 3.2. Host Cell Tolerance to ABLV Infection

**Figure 2:**
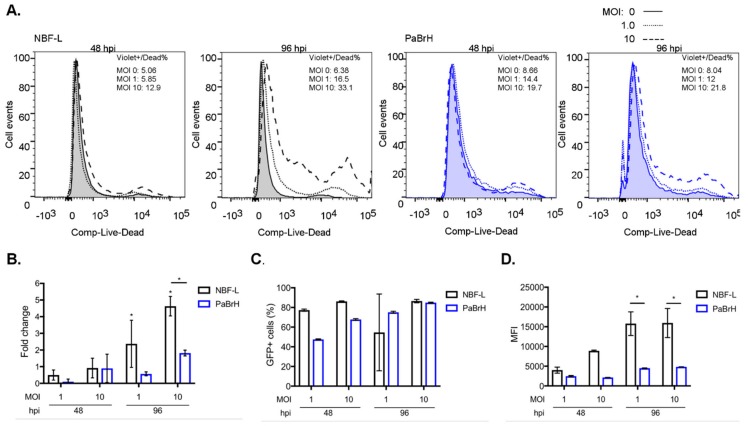
ABLV infection induces less cell death in black flying fox cells. (**A**) Histogram overlays LIVE/DEAD Violet stained NBF-L and PaBrH cells after rABLV-GFP infection. Percentages of Violet^+^ dead cells are demarcated for hpi and MOI indicated. (**B**) Percentage of dead cells expressed as a fold change compared to MOI 0 at each time point. Data are a representative of three independent experiments, * *p* < 0.05, ANOVA (two-way), and Tukey’s multiple comparison test. (**C**) Percentage of GFP positive cells at 48 and 96 hpi. (**D**) Median fluorescence intensity (MFI) of ABLV expressed GFP. Data (**C**–**D**) are representative of two independent experiments, mean ± SEM, * *p* < 0.01, ANOVA (two-way), and Tukey’s multiple comparison test.

We next sought to further investigate the ABLV replication differences between the cells and determine whether black flying fox cells tolerated high doses of ABLV infection. Notably, ABLV infection increased cell death in human and black flying fox cells in a dose-dependent manner (Figure 2A). Black flying fox (PaBrH) cells experienced an increase in cell death at 96 hpi (MOI 10), although a relatively lesser extent compared to human NBF-L cells, which was significantly higher than PaBrH cells at this hpi (Figure 2B). The percentage of GFP positive black flying fox cells was also dose-dependent. Unlike in Figure 1, rABLV-GFP was not washed out after two hpi, which would lessen the effects of inefficient virus entry of black flying fox cells. GFP positive percentages between human and black flying fox cells were comparable with MOI 10 at 48 (~85 vs. 74%) and 96 hpi (~86 vs. 85%), whereas the percentage of GFP positive black flying fox cells was nearly half compared to human cells (~80 vs. 41%) 48 hpi, MOI 1.0 (Figure 2C). More strikingly, a significantly lower vGFP median fluorescence intensity (MFI), which is the quantification of vGFP expression and dependent on post-entry virus replication, was quantified in black flying fox cells compared to a human cell line 96 hpi at both MOI 1 and 10 (Figure 1D). Thus, despite a similar percentage of infected cells at 48 and 96 hpi (MOI 10) in both NBF-L and PaBrH cells (Figure 2C), lower vGFP in PaBrH cells suggested that an intrinsic mechanism, not inefficient entry, restricted production of ABLV encoded proteins (e.g., GFP) or replication.

### 3.3. Infection with ABLV Induced Autophagy

To determine whether an intrinsic defense mechanism was constraining ABLV replication, we next focused on the anti-viral potential of the autophagy pathway. Autophagy functions as an anti-viral mechanism during vesicular stomatitis virus (VSV) infection [55,56]. VSV is a member of family Rhabdoviridae, which includes lyssaviruses such as rabies virus (RABV) and rabies virus-related viruses (e.g., ABLV). Wild-type RABV infection of a human neuroblastoma cell line was shown to activate the autophagy pathway [57]. A hallmark feature of autophagy induction is the cleavage and lipidation of the cytosolic microtubule-associated protein 1A/1B light chain 3B (LC3B-I) to autophagosomal-associated LC3B-phosphatidylethanolamine (LC3B-II). LC3B-II is incorporated into the developing autophagophore and retained in the autophagosome double membrane vesicle such that increased cellular levels of LC3B-II are used as a marker for the induction of autophagy [58]. Functional human and black flying fox LC3B proteins are identical in amino acid sequence after post-translational cleavage. Therefore, we compared quantities of LC3B-II between black flying fox and human cell lines by immunoblotting, following inhibition and stimulation of autophagy.

**Figure 3:**
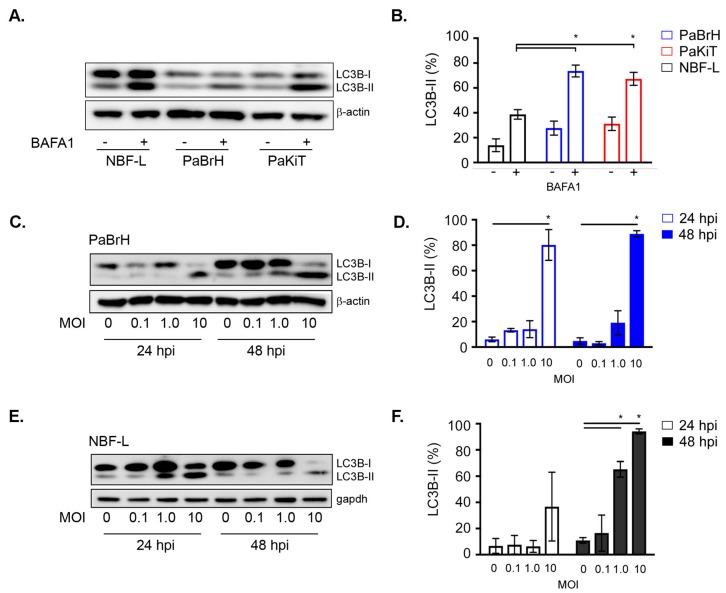
ABLV infection activates autophagy. (**A**) LC3B western blot of lysates from human NBF-L and black flying fox PaBrH and PaKiT cells, treated with BAFA1 (400 nM; 2 h). (**B**) LC3B-II (β-actin-normalized) from BAFA1 treated cells. (**C**) LC3B western blot of lysates from PaBrH cell lysates. PaBrH cells were infected for times (hpi) and MOI indicated. (**D**) LC3B-II (β-actin-normalized) expressed as a percentage of total LC3B from PaBrH cells infected with rABLV-GFP. (**E**) LC3B western blot of lysates from NBF-L cells infected with rABLV-GFP. (**F**) LC3B-II (gapdh-normalized) from NBF-L cells infected with rABLV-GFP. PaBrH and NBF-L cells were infected with rABLV-GFP for times (hpi) and MOI indicated. All data are a representative of three independent experiments, LC3B-II is expressed as a percentage of total LC3B, mean ± SEM, * *p* < 0.05, student’s *t*-test.

Bafilomycin-A1 (BAFA1) inhibits autophagic flux by blocking the fusion of autophagosomes and autolysosomes [59,60], causing a buildup of autophagosomes and LC3B-II. We treated PaBrH, PaKiT, and NBF-L cells with BAFA1 for two hours to inhibit autophagic flux and create a snapshot of basal autophagy levels in all three cell lines. As expected, BAFA1 treatment significantly increased levels of LC3B-II in all three cell lines (Figure 3A,B). Interestingly, in the BAFA1 treated cells, levels of LC3B-II were significantly higher in both PaBrH and PaKiT compared to NBF-L (Figure 3A,B), suggesting that these black flying fox cell lines maintained a higher basal level of autophagic flux.

**Figure 4:**
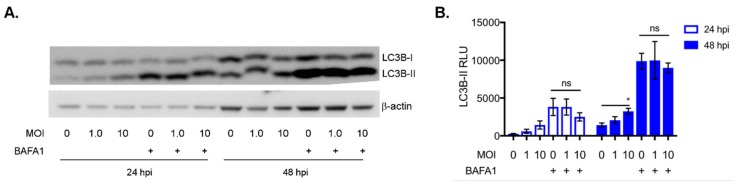
ABLV infection and autophagy activation in primary black flying fox brain cells. (**A**) Western blot for LC3B in primary black flying fox brain (PaBr) cells following 24 and 48 hpi with wt-ABLV MOI indicated. BAFA1 treatment (400 nM) was used as a control for blocking autophagic flux. (**B**) Quantification of LC3B-II levels (β-actin normalized) relative light units (RLU) from cells infected in (**A**). All data are representative of three independent experiments, mean ± SEM and * *p* < 0.05 ANOVA (two-way), and Tukey’s multiple comparison test (48 hpi MOI 0 vs. MOI 10).

To determine whether autophagy was activated in bat versus human cells after ABLV infection, we again monitored levels of LC3B-I and LC3B-II. PaBrH cells showed a significant percentage of LC3B-II conversion when infected with a MOI 10 at 24 and 48 hpi (Figure 3C,D). The NBF-L cells had a significant percentage of LC3B-II when infected for 48 hpi (MOI 1.0 and 10) (Figure 3E,F). We confirmed these results in primary black flying fox brain (PaBr) cells. A significant increase in LC3B-II was again dependent on a high virus dose, MOI 10 (Figure 4A,B). Additionally, treatment with BAFA1 increased LC3B-II, however the levels of LC3B-II remained statistically equivalent regardless of ABLV MOI. As there was not an additive effect of ABLV infection plus BAFA1 treatment, it is possible that the basal level of autophagic processes are so rapid that external stimulation (a) has little observable effect by comparison or (b) additional stimulation via ABLV infection increased autophagic flux such that autophagosome and LC3B-II turnover occurs more rapidly. These results suggested that although ABLV induced autophagy in both black flying fox and human cells, further enhancement of autophagic flux in black flying fox cells required a high virus dose. This observation highlights the need for additional studies of autophagic kinetics, both basal and induced, in black flying fox cells.

### 3.4. ABLV does not Inhibit Autophagic Flux

**Figure 5:**
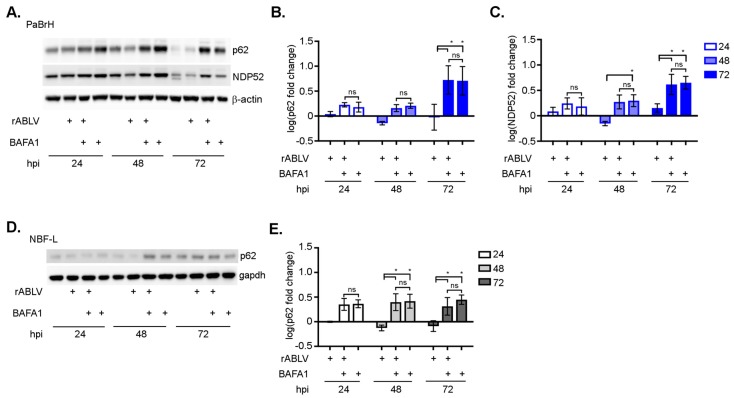
ABLV infection does not inhibit autophagic flux. (**A**) Western blot for p62 and NDP52 proteins in PaBrH cell lysates, rABLV-GFP (MOI 1.0), and BAFA1 (400 nM; 2 h). (**B**) Log transformed fold change of p62 protein (PaBrH) normalized to β-actin and compared to uninfected DMSO-mock treated time point controls. (**C**) Log transformed fold change in NDP52 protein (PaBrH) normalized to β-actin and compared to uninfected DMSO-mock treated time point controls. (**D**) Western blot for p62 in NBF-L cell lysates. (**E**) Log transformed fold change in p62 (NBF-L) normalized β-actin and compared to uninfected DMSO-mock treated time point controls. All data are representative of three independent experiments, mean ± SEM, * ANOVA (two-way), and Tukey’s multiple comparison test.

To further characterize the increase in LC3B-II after ABLV incubation, we monitored cytosolic levels of autophagy cargo receptor proteins, p62 and NDP52, which are associated with selective autophagy and used to monitor autophagic flux [61,62,63,64]. Virus proteins, such as HIV Nef protein and influenza M2, inhibit autophagic flux by blocking fusion of autophagosomes with autolysosomes [65,66]. Inhibition of autophagic flux and the resulting accumulation of autophagosomes and their contents such as LC3B-II could be misinterpreted as virus activation of autophagy. If autophagic flux is unobstructed by ABLV infection, p62 and NDP52 will be degraded, whereas inhibition of autophagic flux will lead to an increase in these cargo proteins. We examined levels of p62 and NDP52 in PaBrH cells after ABLV infection, and in combination with BAFA1 treatment. Infection with ABLV alone resulted in less p62 (Figure 5A,B) and NDP52 (Figure 5A,C) detected in PaBrH cells at 48 hpi. In NBF-L cells, a mean fold decrease in p62 was observed at both 48 and 72 hpi (Figure 5D,E). Treatment with BAFA1 alone or in combination with ABLV infection at all time points resulted in a significant increase of p62 and NDP52 in PaBrH cells (Figure 5A–C) and NBF-L cell (Figure 5D,E), however p62 and NDP52 fold-changes between ABLV alone and ABLV plus BAFA1 were insignificant. Since additive effects of BAFA1 inhibition of autophagic flux were not observed during ABLV infection and we observed a fold decrease of p62 and NDP52 48 hpi, we concluded that ABLV alone was not inhibiting autophagic flux. Together, increased LC3B-II (Figure 3 and Figure 4) and decreased p62/NDP52 protein levels (Figure 5) in black flying fox cells further demonstrated that ABLV infection induced the autophagy pathway.

### 3.5. Anti-Viral Role of Autophagy during ABLV Infection

**Figure 6:**
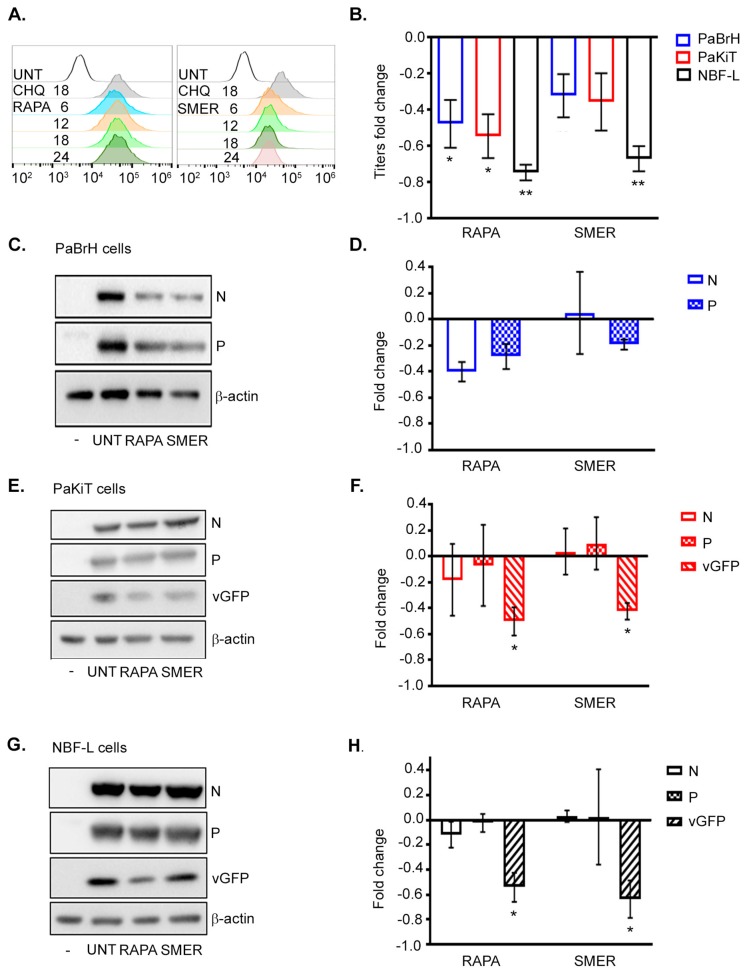
Pharmacologic activation of autophagy limits ABLV replication. (**A**) Flow cytometry histogram overlays of PaKiT cells stained with CYTO-ID^®^. PaKiT cells were treated with RAPA (2 µM), and SMER (50 µM) for h indicated; as well as CHQ (1 µM, 18 h). (**B**) Fold change in rABLV-GFP titers (pfu/mL) fold change from PaBrH, PaKiT, and NBF-L cell culture supernatants collected 48 hpi (MOI 1.0). Cells were treated with RAPA (2 µM) and SMER (50 µM) for 24 h. (**C**) Western blot of ABLV N, P, and vGFP in PaBrH cell lysates. (**D**) Fold change in ABLV N, P, and vGFP proteins (β-actin-normalized) from PaBrH cell lysates. (**E**) Western blot of ABLV N, P, and vGFP collected in PaKiT cell lysates. (**F**) Fold change in ABLV N, P, and vGFP proteins (β-actin-normalized) collected from PaKiT cell lysates. (**G**) Western blot of ABLV N, P, and vGFP collected in NBF-L cell lysates. (**H**) Fold change in ABLV N, P, and vGFP proteins (β-actin-normalized) collected from NBF-L cell lysates. (**C**–**H**) Whole cell lysates were collected 48 hpi (rABLV-GFP MOI 1.0). Fold change is a representation of at least three independent experiments, mean ± SEM and ** *p* < 0.001, * *p* < 0.05 ANOVA (one-way) analysis, Dunnett’s multiple comparison test with treatments compared to DMSO mock treated cells.

To understand whether the autophagy pathway had an anti-viral role during ABLV infection, we examined the effects of pharmacological activation of autophagy on ABLV replication. The mammalian target of rapamycin (mTOR) is a key modulator of autophagy [67]. We therefore tested the effects of both mTOR-dependent autophagy activation using the mTOR inhibitor rapamycin (RAPA), as well as mTOR-independent autophagy activation with small molecule enhancer of autophagy-28 (SMER) stimulation [68,69]. rABLV-GFP infected black flying fox cell lines, PaBrH and PaKiT, were treated with RAPA or SMER, respectively, during ABLV infection to elucidate whether activation of autophagy had anti-viral effects; human NBF-L cells were treated the same in comparison. RAPA and SMER activation of autophagy was monitored by CYTO-ID^®^ (Enzo) staining of autophagosomes in PaKiT cells. Treatment with either RAPA or SMER induced autophagy in PaKiT cells, with RAPA showing a stronger effect (Figure 6A). Chloroquine (CHQ), an inhibitor of autophagic flux, was included as a positive control for autophagosome staining [60]. Treatment of black flying fox and human cells with RAPA after ABLV infection resulted in significant reductions in ABLV replication (Figure 6B). A significant reduction in ABLV titers after SMER treatment was only observed in human NBF-L cells, and a decreasing titer trend was observed in SMER treated black flying fox cells.

As a potential mechanism underlying the reduction in ABLV, we examined whether treatment with RAPA or SMER resulted in degradation of virus proteins. Cell lysates collected after RAPA and SMER treatment of PaBrH and PaKiT cells, followed by analysis via western blot (Figure 6C–F). Neither RAPA or SMER treatment had any significant effects on N or P protein levels in PaBr cells, however, N protein fold change had a decreasing trend in RAPA treated cells (Figure 6C,D). ABLV protein levels in bat PaKiT cells, infected and treated with RAPA and SMER, were examined by western blot (Figure 6E,F). As seen in the PaBrH cells, N protein fold change had a decreasing trend in PaKiT cells when treated with RAPA, but not SMER. P protein levels were quantified for two independent experiments, and also had a trending decease in both RAPA- and SMER-treated cells. Although, western blot quantification of vGFP can be difficult because of low replication and vGFP expression levels in bat cells, we successfully quantified vGFP from two of the four independent experiments with PaKiT cells. The noticeable vGFP fold decrease caused by RAPA and SMER stands in contrast to fold changes in N and P proteins. Human NBF-L cells had a significant fold decrease of vGFP levels in both RAPA- or SMER-treated cells, but neither N nor P levels had any significant reductions (Figure 6G,H). These results indicated that autophagy acts as an anti-viral response in both black flying fox cells and human cells during ABLV infection. Given our limited on-hand reagents (e.g., ABLV N and P reactive antisera), we were only able assess the effects of autophagy activation on N and P protein levels. It is possible that other ABLV proteins such as M protein or L RNA polymerase, to which we lack reagents, are degraded by autophagy, and that degradation of these proteins leads to decreased ABLV replication as assessed by lower ABLV titers (Figure 6B) and ABLV vGFP expression (Figure 6F,H).

### 3.6. NVP BEZ235 Treatment Restricts wt-ABLV Replication

**Figure 7:**
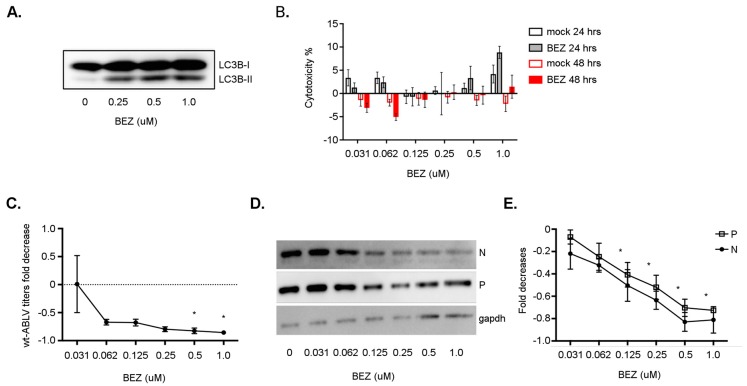
BEZ treatment reduced wt-ABLV replication in a human neuroblastoma cell line. (**A**) Western blot of LC3B protein in lysates from NBF-L cells were treated with indicated concentrations of BEZ for 4 h. (**B**) Lactate dehydrogenase cytotoxicity assay of NBF-L cells treated with BEZ or mock-treated with DMSO. ANOVA analysis revealed no significant differences of quantified spontaneous cytotoxicity between DMSO or BEZ treatments. (**C**) Fold decreases in wt-ABLV titers (pfu/mL) from cells treated in (**A**) fold decreases. (**D**) Western blot image of wt-ABLV N and P protein in NBF-L lysates prepared in (**A**). (**E**) Fold decreases in wt-ABLV N and P protein from cells treated in (**A**). Data are a representative of three independent experiments, mean ± SEM, * *p* < 0.05 ANOVA (one-way) analysis BEZ (µM) compared to DMSO (mock) treatment.

While conducting this study we observed that autophagy activation through pharmacological modulators (e.g., RAPA) reduced ABLV replication and protein expression in a human neuroblastoma cell line (NBF-L). Infected neurons cannot rely on the anti-viral effects of IFN-induced cell death to control viral infection and risk potential damage to the central nervous system [70]. Given the neurotropism of lyssaviruses such as ABLV and RABV, we asked whether potent autophagy induction with dactolisib (a.k.a. NVP BEZ235 or BEZ), a dual inhibitor pan-class PI3K and mTOR inhibitor evaluated in clinical trials, could also restrict ABLV replication in NBF-cells. Treatment of NBF-L cells with BEZ increased the amount of autophagosomal LC3B-II indicative of autophagy activation (Figure 7A) without causing significant cytotoxicity (Figure 7B). To examine the anti-viral effect of BEZ treatment on ABLV replication, NBF-L cells were infected with wt-ABLV for 24 h then treated with BEZ. Treatment with BEZ resulted in significant dose-dependent decreases of wt-ABLV titers (Figure 7C), in addition to N and P protein levels (Figure 7D,E). Although BEZ advanced to Phase I/II clinical trials as a potential anti-cancer therapy [71,72,73], its use was discontinued due to adverse toxicities [74]. Nevertheless, our results suggest that with improved selectivity or dosing, potent inducers like BEZ may be promising additional therapeutics to prevent lethality following lyssavirus exposure.

## 4. Discussion

Although bats are reservoir hosts for a richness of viruses, a comprehensive understanding of how their immune response can sense, respond to, and control viral infection is needed. The type I IFN pathway is well-conserved among vertebrates as an intrinsic first defense during virus infection, with unique anti-viral expression patterns noted in bat cells [75]. In black flying fox cells, IFN-α was constitutively expressed regardless of virus infection, whereas IFN-β was not induced [50], consistent with another report that IFN-β was antagonized during HeV infection [45]. Regardless, the IFN pathway is a constant target of virus antagonism and evasion.

Here, we focused on another well-conserved, intrinsic immune response and present a first experimental attempt to study the autophagy pathway during a virus infection in cell lines derived from a bat species. Collectively, our findings demonstrated that ABLV infection increased autophagosome generation and autophagy in black flying fox and human cells, and that autophagy activation in black flying fox cells was dependent on a high ABLV MOI. Yet, how ABLV RNA or proteins are sensed by host sensory machinery during ABLV replication remain unanswered. A related rhabdovirus, VSV, induces autophagy through G protein engagement independent of virus replication [56] and RABV M protein may also contribute to autophagy activation [57]. Autophagy has been implicated in either anti- and pro-viral processes for distinct viruses [33,76]. For example, a protective role for autophagy was identified during VSV infection [55,56]. In our study, pharmacological activation of autophagy decreased ABLV replication in both black flying fox and human cell lines, suggesting autophagy antagonizes lyssavirus infection.

Host–virus adaptations that promote tolerance to virus infection may contribute to the ability of bats to act as primary animal hosts without developing pathogenic disease [77]. We found that the black flying fox brain cell line did not undergo significant cell death after what could be considered a pathogenic ABLV infection (MOI 10; 96 hpi). In contrast, ABLV induced substantial cell death in human neuroblastoma cells, similar to that observed previously with RABV infection [57]. Crosstalk and interplay between autophagic and apoptotic processes have long been recognized. Dependent on the environment or stimulation, autophagy can promote survival under stressful conditions (e.g., nutrient deprivation) or itself act as a mechanism of programmed cell death if left unchecked [78,79]. Recently, we rescued a recombinant Cedar virus [80], a non-pathogenic *Henipavirus* species also isolated from *Pteropus* species [81]. Such tools will facilitate opportunities to ask if autophagic response to ABLV can be generalized to other bat-borne viruses, and better define how the balance between autophagy and cell death is calibrated in bat cells.

The interactions between natural reservoirs hosts and viruses require that (a) the host is readily infected and (b) the virus needs to persist in the host long enough to be transmitted to another susceptible host, before it is either cleared by the host immune response or kills the host [82,83]. Epidemiology models suggest a possibility that latently infected individual bats contribute to episodic shedding and natural persistence of bat-borne viruses [84]. Although horizontal transmission within a population is still regarded as the primary source of transmission [85], HeV and NiV recrudescence in *Pteropus* hosts have been reported [86,87,88], yet the precise mechanism of latency or persistence is unclear. Autophagy functions as a pro-survival defense by removing toxic virus N protein aggregates during Sindbis virus infection, without having an effect on virus replication [89,90]. Indeed, a pro-survival autophagic response that removes toxic protein aggregates and dampens virus replication might be critical for promoting viral persistence at the cellular level in natural hosts, maintaining the potential to shed virus with minimal disease presentation. More investigation is needed to translate in vitro bat immunity studies into a models of virus persistence and shedding within individuals and populations.

In conclusion, our working hypothesis is that black flying fox cells maintain a higher basal rate of autophagy that serves as both an anti-viral and pro-survival mechanism when further stimulated by virus infection. Similarly, Brook and Dobson, 2015, postulated that autophagic removal of damaged mitochondria (i.e., mitophagy), a result of enhanced oxidative stress from the demands of flight, evolved a secondary anti-viral role for autophagy in bats [91]. New cell lines have recently been derived from additional bat species facilitating a greater comparative study across the bat taxa [92]. Furthermore, comparisons with rodent cell lines will strengthen whether in vitro intrinsic responses to virus infection are unique to bats or shared co-adaptations among other natural host species. Experimental challenges have limited our efforts to genetically silence or pharmacologically inhibit the autophagy pathways in black flying fox cells. Although we cannot yet definitively associate autophagy function with host tolerance to ABLV infection in black flying fox cells, ongoing experiments will refine our mechanistic understanding of enhanced basal autophagy and virus-induced autophagy in bat cells, and further characterize the pro-survival role of autophagy during virus infection.
